# Protocol for the measurement and analysis of human photoencephalographic signals

**DOI:** 10.1016/j.xpro.2025.104223

**Published:** 2025-12-04

**Authors:** Mattia Bonzanni, Nicolas Rouleau, Nirosha J. Murugan

**Affiliations:** 1 Department of Pediatrics, Weill Cornell Medicine, New York, NY 10066, USA; 2 Department of Health Sciences, Wilfrid Laurier University, Waterloo, ON N2L 3C5, Canada; 3 Department of Biomedical Engineering, Tufts University, Medford, MA 02155, USA; 4 Allen Discovery Center, Tufts University, Medford, MA 02155, USA

**Keywords:** Biophysics, Cognitive Neuroscience, Energy

## Abstract

Photoencephalography, involving the measurement of ultraweak photon emissions (UPEs), is increasingly being recognized for its potential significance in understanding brain activity. We present a protocol for recording, analyzing, and interpreting UPE signals in humans. We outline the steps for simultaneous measurement of UPEs and electric fields around the head, using photomultiplier tubes and electroencephalography, as well as data processing and interpretation. This protocol guides the emerging field of biophotonics, establishing a structured pipeline for UPE acquisition and analysis in humans.

For complete details on the use and execution of this protocol, please refer to Casey et al.^1^

## Highlights

• Instruction for concomitant photoencephalography and electroencephalography in humans
• Steps for ultraweak photon emission data analysis
• Guidance on statistical analysis of photoencephalographic data

## Abstract (editor highlights)

Publisher’s note: Undertaking any experimental protocol requires adherence to local institutional guidelines for laboratory safety and ethics.

## Graphical abstract

**Figure:**
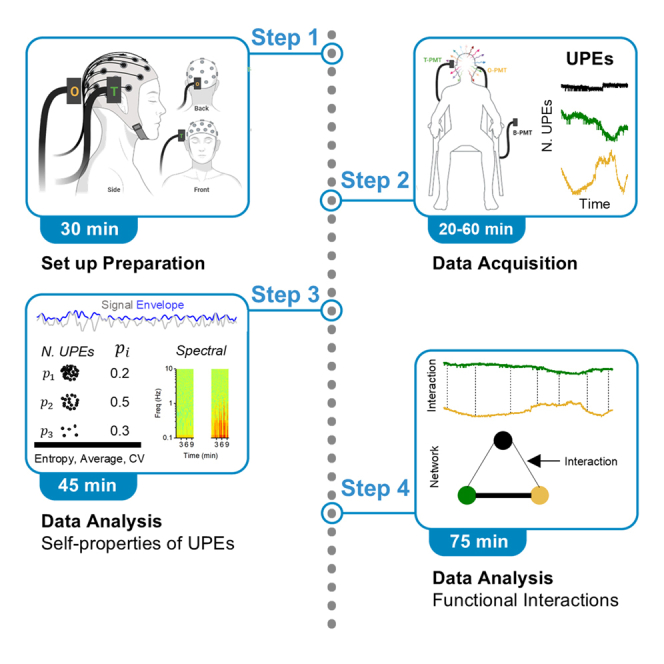

## Before you begin

While ultraweak photon emissions (UPEs) have been increasingly recognized as a potential source of biological information, their origin and functional significance remain under investigation.^2^ UPEs may arise from bioluminescent reactions linked to oxidative stress, mitochondrial function, or even quantum effects in neurons.^2^ Despite this uncertainty, advancement in technologies such as photomultiplier tubes (PMT), single-photon counters (SPC), and charge-coupled devices (CCD) allowed to record UPEs across different modalities, including the brain, skin, cell cultures, *ex vivo* tissues and plant cells.^2^^,^^3^^,^^4^^,^^5^ UPEs recorded from human brains have been hypothesized to correlate with neural activity and cognitive processes^6^^,^^7^^,^^8^; however, conclusive evidence has remained elusive. To better understand how UPEs relate to brain function, photodetectors must be integrated with traditional neuroimaging technologies for simultaneous measurement of brain light emissions and known correlates of neural activity (e.g., electric fields, magnetic fields, metabolic activations, blood (de-)oxygenation). If UPEs are found to track and/or predict reliable correlates of neural activity, the functional relevance of light emissions from the brain can be elucidated.

This protocol outlines a step-by-step process to set up recording devices, as wells acquire and analyze UPEs from human brains – a technique termed “photoencephalography” – simultaneously with electroencephalography (EEG) neuronal signals. UPE traces are analyzed for their temporal similarity (hierarchical clustering), descriptive properties (mean and coefficient of variation), entropy, regulatory complexity (Detrend Fluctuation Analysis scaling exponent), spectral properties (short Fourier Transformation and multitaper analysis), time synchronicity (correlation), frequency synchronicity (coherence), causal influence (Granger Causality) and information flow (Mutual Information). Typically, preparation and data acquisition will require 1–1.5 hours. An additional 1-2 hours are expected for data analysis. Therefore, the total time of the protocol is 2–3.5 hours; however, timing associated with the experimental design can be quite variable.

### Innovation

This protocol establishes a structured pipeline for the measurement, analysis, and interpretation of UPE data from human brains, termed photoencephalography. It also integrates PEG with a conventional neuroimaging technique, EEG, which can be performed simultaneously.

### Download scripts

1. Download files from Zenodo: https://doi.org/10.5281/zenodo.15830349. This folder contains the following files (script + example dataset):

### Institutional permissions

All human experiments were performed according to the Tri-Council Policy Statement (TCPS 2): Ethical Conduct for Research Involving Humans with permission from the Institutional Research Ethics Board at Algoma University (Study Approval Number: 026-202-122).

### Preparation of experimental setup

2. Prepare the experimental room:
  a. Choose a recording space or chamber that enables the control of ambient light conditions and electrical noise while maintaining air exchange for human participants.
    ***Note:*** Ideally, recordings should be obtained within a darkened Faraday cage to reduce external interferences of electromagnetic source.
  b. Line the walls of the chamber with dark foam blocks, black curtains or non-reflective black paint with a light reflectance value as close as possible to zero to minimize reflective surfaces.
    ***Note:*** Be mindful not to block vents for air exchange. No external sources of heat should enter the room; however, cold air can be introduced if necessary. Fans should not be present in the room because they are both a source of heat and electrical noise. Seasonal variations and building HVAC control must be monitored and controlled where possible.
  c. Place a comfortable chair that does not swivel, rotate, or recline in the center of the room (preferably black in color), where participants will sit during the recordings.
  d. Run USB cables for individual PMTs and the EEG amplifier box from the center of the experimental room to an external room or antechamber via conduits or ports that enable data exchange with the doors of the chamber closed.
    ***Note:*** While specialized ports may be impermeable to light, any open conduits should be optically shielded with brush or rubber seals.
    ***Note:*** For cable management, assign labels to each cable using small pieces of tape. In addition, to reduce potential electrical noise sources, the room should be equipped with a grounding system with low impedance, with ground loop isolators or circuit breakers for each set up potentially needed.
  e. Set up as many computers as needed for the experiment and plug in corresponding USB cables.
    ***Note:*** In the original study,^1^ to more easily manage multiple graphical user interfaces associated with recording software, we used independent laptop computers for EEG and PMT devices.
3. Prepare EEG system:
  a. Position EEG digital amplifier box behind the chair with all external LEDs removed or blocked to eliminate sources of light within the chamber.
    ***Note:*** A small piece of black vinyl (electrical) tape will obscure LEDs from the amplifier, which can be confirmed using a PMT (adding layers of tape until fully shielded.
  b. Choose an appropriate montage, which refers to the arrangement and reference configuration of electrodes used to record brain activity.
    ***Note:*** The selected montage should align with the goals and requirements of the experimental design, such as the spatial resolution needed, the brain regions of interest, and the type of neural activity being investigated. Some commonly used montages include:
    i. Referential montage - all electrode signals are referenced to a single, electrically neutral site (e.g., linked ears, mastoid, or vertex), which emphasizes the spatial distribution of activity relative to that reference point.
    ii. Bipolar montage - adjacent electrodes are paired to measure voltage differences between nearby sites, which is useful for identifying localized activity or gradients.
    iii. Average reference montage - signal at each electrode is referenced to the average of all electrodes, which helps reducing noise while focusing primarily on relative activity across the scalp without dependence on a single reference site.
    iv. Laplacian montage - each electrode is referenced to the average of its immediate neighboring electrodes, enhancing spatial resolution by emphasizing local activity.
      ***Note:*** In our original study,^1^ we used a referential montage, referenced to the ears. This type of montage is widely used in EEG research because it preserves the spatial distribution of brain activity relative to a relatively neutral reference site, allowing for clearer interpretation of voltage topographies across the scalp. By referencing the ears, we minimized contamination from focal cortical activity and ensured that the recorded signals reflected genuine neural activity over the cortical regions of interest. Select electrodes were (Fp1, Fp2, F7, F3, Fz, F4, F8, T3, C3, Cz, C4, T4, T5, P3, Pz, P4, T6, O1, O2), ear references (A1, A2), and ground electrodes (also on the ears, Az).
  c. Set sampling rate and bandpass filters for data collection.
    ***Note:*** These parameters must be properly selected to capture the full range of neural activity relevant to the study’s objectives. The sampling rate should be at least two-three times the highest frequency of interest to avoid aliasing (which is the misrepresentation of high-frequency signals as lower frequencies due to insufficient sampling rate). In addition, bandpass filters are applied to limit the recorded signal to specific frequency bands of interest, while excluding unwanted noise or artifacts such as electrical interference (e.g., 50/60 Hz line noise).
    ***Note:*** In our original study,^1^ we use a sampling rate of 250 Hz, low cut filter of 1.6 Hz, and a high cut filter of 50 Hz, and notch filters of 50-70 Hz and 110-130 Hz to minimize noise. Gain was set to 100 μV.
  d. Connect a 19-channel electro cap to the amplifier box in the chamber, ready to be applied to the participant’s head.
    ***Note:*** Other montages can be used as needed (e.g., to increase spatial resolution of EEG recordings, increase electrode density); however, spaces between electrodes must remain sufficiently available to include the placement of PMTs with unobstructed lines of sight to the head.

4. Prepare PMT/UPE system:
  a. Position flexible mounts around the chair at the center of the recording room.

***Note:*** Mounts can be clamped to the chair or supports positioned around the chair.

5. Insert PMTs into flexible mounts (e.g., gooseneck phone holder mount), orienting apertures toward the center of the chair:

***Note:*** The PMT’s aperture must be closed at all times other than during recording periods to minimize dark counts and extend the device’s functional lifespan. We used PMTs with a spectral range of 280–850 nm, rated for dark counts of approximately 1000 photons s^–1^. A 22-mm S20 cathode within the PMT served as the primary sensor. In general, users should select PMTs or other photodetectors with low dark counts, low dark count variability, high quantum efficiency, larger apertures, and broad spectral ranges that include UV, visible, and near-IR ranges.

6. Label all PMTs, matching labels on data-transmitting USB cables:

***Note:*** In our original study, we used three PMTs: Background (B-PMT), left occipital lobe (O-PMT), and right temporal lobe (T-PMT). The B-PMT was always placed on the left side of the participant, which was the further point from the door to the room, with the aperture pointed away from the body, toward a wall. While other positions may be valid, the B-PMT should always be pointed away from the participant or any other potential light source with an open, unobstructed aperture.

7. Provide external power to PMTs:

***Note:*** The power supply (5 V input voltage) may interfere with EEG recordings. Therefore, cable management is critical. Do not cross power supply cables with any EEG-related equipment. To minimize cross-talk and other sources of electrical noise, cables should not be bundled or twisted.

8. Set recording parameters (in software, e.g., Sense-Tech Counter Timer Software) of the PMTs to accommodate the experimental design:

***Note:*** We selected a sample rate of 25 Hz (40 ms intervals) with a high-cut filter of 30 Hz, notch-filter of 60–120 Hz and gain of 100 μV. Recordings were set to automatically terminate after 15,000 measurements, or 10 minutes sampling at 25 Hz.

### Welcoming the participant

**Timing: 5 min**

Participants are greeted, consent is obtained, and instructions are provided to guide the measurement procedures.

9. Greet the participant and provide a description of the experimental procedure and its scope.
10. Obtain informed consent.
11. Provide instructions for the participant:
  a. Direct the participant to the chair in the recording chamber.
  b. Explain that the devices will be placed on and around their head.
  c. Answer all of the participants’ questions as necessary.

### Setting up the EEG

**Timing: 30–40 min**

Electroencephalographic equipment, including the Electro-cap and references, must be applied and tested for impedance and electrical artifacts.

**CRITICAL:** Preparation of EEG must be careful (see Notes on gel application technique, referencing, impedance, and common sources of noise) so as to minimize artifacts and maximize signal-to-noise ratio.

***Note:*** Here, we describe an EEG configuration with passive electrodes. Because UPEs will be measured simultaneously, we reasoned that active electrodes with localized electric fields in close proximity to the PMTs might contribute to spurious photon measurements due to interference or distortion of detection.

12. Display the Electro-Caps, blunted syringes, and gel for the participant to see.

***Note:*** It is important to clearly demonstrate that the syringe is blunt to eliminate the expectation of being poked by a sharp object.

13. Select cap size based on the participant’s head size (e.g., small, large).
14. Apply Electro-Cap to participant’s head and pull down to secure firmly and proceed to plug the cap’s cable into the amplifier box to begin streaming data.

***Note:*** Do not plug the cap into the amplifier box until the cap is already on the participant’s head and in the correct position.

15. Apply gel to each sensor by inserting the gel-filled, blunted syringe into the holes arranged throughout the cap according to the 10–20 International System of Electrode Placement.

***Note:*** Gel must form a conductive bridge between the participant’s scalp and the electrode’s recording surface.

***Note:*** If the participant has voluminous hair, gently twirl the syringe to painlessly penetrate toward the scalp.

***Note:*** It is good practice to inject gel into the space between the electrode and scalp as the syringe is being pulled up toward the surface, which provides an even distribution.

16. Once gel has been applied to each electrode, check impedance values in the recording software (e.g., WinEEG). Values less than 5 kOhm are considered appropriate for recordings. Adjust gel volume if necessary.

***Note:*** Too much gel can cause abnormal measurements because it may create electrical bridges between adjacent electrodes. Removing gel with a paper towel is possible; however, it is best to gradually add small amounts rather than over-injecting gel.

17. Apply a small amount of gel to ear-clip electrodes and affix them to the participant’s ears. Plug each electrode into the amplifier box as references for the monopolar montage.

***Note:*** If the participant is wearing removable piercings on the earlobes, removing them is ideal.

18. Assess signal quality by recording for a short period.

***Note:*** Make note of 60 Hz buzz, abnormal DC shifts, or significantly distorted signal. Troubleshoot if necessary, checking connections, gel volume, reference placement, grounding, and placement of electrical equipment in the room.

### Setting up the PMTs

**Timing: 5 min**

Photomultiplier tubes must be positioned around the head and the counter software must be initialized before darkening the room in preparation for measurements.

**CRITICAL:** PMTs must be shielded from direct light (e.g., light introduced by experimenters to visually navigate in the room and complete setup) at all times to avoid high dark counts which can interfere with UPE measurements. Shielding can be accomplished by taping a thin, opaque piece of plastic over the aperture when the device is not in use and storing the PMTs in anti-static, opaque bags. Background, ambient recordings should always be included in the experimental design to account for variations within the room itself and incorporated in analyses (see data analysis section).

19. Position PMTs next to the head by adjusting the clamped, flexible mount.

***Note:*** The aperture should be oriented toward the head, positioned within 5 cm of the surface of the head and Electro-Cap, though never so close as to make physical contact with the hair or scalp. The line-of-sight of the PMT to the head should not be obstructed by electrodes.

***Note:*** PMT positioning should be consistent between subjects. If the device is positioned too close to the head, there is a chance that unintentional movements by the participant will result in collisions with the device, possibly affecting recordings.

20. Initialize Counter Timer software, inputting parameters discussed in “Preparation of experimental setup”. Set recording configuration to terminate after X measurements at the chosen sampling rate.
21. Wait for a minimum of 5 minutes after closing the chamber doors for dark counts within the recording space to return to stable, minimal levels.

**Figure 1:**
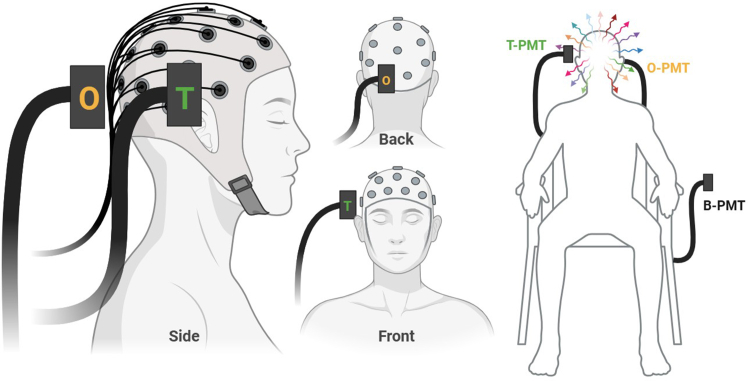
Equipment setup Example of EEG and PMT devices configured and oriented to record simultaneous electric potentials and UPEs from the head. Lateral (Left), posterior (Middle, Top), anterior (Middle, Bottom), and full-body (Right) views are represented. The EEG cap electrodes are referenced to ear clip electrodes (not shown). The current configuration includes two PMTs around the head, positioned over the left occipital (O) and right temporal (T) regions, corresponding to spaces adjacent to EEG sensors O1 and T4, respectively. A third PMT (B-PMT) is placed in the same space, away from the participant to record background light measurements. Note that participants should be sitting in a darkened and electromagnetically shielded recording space during recordings. This figure was partially created with BioRender.

Figure 1 showed the equipment setup.

## Key resources table

**Table:** 

REAGENT or RESOURCE	SOURCE	IDENTIFIER
**Deposited data**		

https://doi.org/10.5281/zenodo.15830349	Zenodo	zenodo.15830349

**Software and algorithms**		

MATLAB	MathWorks	https://www.mathworks.com/products/matlab.html
R	R Project	https://www.r-project.org/
WinEEG	MITSAR	https://mitsar-eeg.com/product/wineeg-advanced/
Counter Timer	Sens-Tech	

**Other**		

Mitsar-202 QEEG amplifier	Bio-Medical, Inc.	#NTE MITSAR20224
19-channel Electro-Caps	Bio-Medical, Inc.	#ECA CAPS
Electro-gel for Electro-Caps	Bio-Medical, Inc.	#ECA E9
BD Luer-Lok tip syringes, 5 mL	Bio-Medical, Inc.	#ECA E7B-5ML
Electro-Cap tin ear electrodes, 9 mm	Bio-Medical, Inc.	#ECA E5-9S
Photomultiplier tubes	Sens-Tech	DM0090C
Flexible (gooseneck) mount/clamp	Lamicall	LS05-CA-B

## Step-by-step method details

### Data acquisition

#### Acquisition of ultraweak photon emissions and electroencephalography data across tasks

**Timing: 5 min**

**Test EEG signal quality:** EEG signal quality must be tested by inspecting for electrical artifacts, identifying expected brain-based signals, and evaluating responsiveness to participants’ actions in real time.

To test EEG signal quality, initialize a measurement in WinEEG and look for conspicuous artifacts and other anomalous signals. Assuming the integrity of individual sensors and an adequate salt bridge between the gel and scalp of the participant, the most common sources of noise within an EEG signal is the 60 Hz buzz from local power supplies (in North America) and significant DC shifts from reference electrode displacement. Grounding equipment appropriately and adjusting ear references typically resolves most issues. To ensure responsiveness of the system, it is common practice to examine the EEG record when the participant opens and closes their eyes, where the latter is likely to produce significantly increased alpha rhythms (∼10 Hz on visual inspection) in the posterior regions of the cerebrum with a generalized low-frequency oscillation gradient that fades toward the anterior pole of the brain. Other methods of assessment include asking participants to blink or clench their jaw, generating real-time changes to the record.

**Test PMT signal quality:** PMT signal quality must be tested by checking dark counts while identifying potential drift or signal stabilization in the darkened room.

**Timing: 5 min**

To test PMT signal quality, initialize a measurement in the Sens-Tech Counter Timer software and examine dark count magnitude. Values should track manufacturer specifications; however, dark counts can change over time and may be compared to previous measurements or known minima for the specific device being used in the recording space or chamber. A slight drift toward increased dark counts over time could indicate that the recording chamber is becoming warm. Therefore, direct measurement of the room’s temperature with a digital thermometer is recommended for reporting (and, if necessary, a covariate for statistical analyses). Stable PMT signals can often be achieved by waiting for some time at the beginning of experiments for the room to become dark.

**Initiating simultaneous recording:** Simultaneous recording of EEG and PMT signals is initiated. Manual synchronization of signals will produce an offset of the time series data; however, this can be addressed later by averaging values across temporal bins. Ideally, specialized hardware or software with triggers and timestamping features should be used to ensure synchronization.

**Timing: Variable, depending on the task**

A typical EEG-PMT data acquisition session may involve anywhere between 20 mins and 1 hour of continuous measurement; however, timing will depend on experimental design.

### Data analysis

#### Create dataset

**Timing: 5–10 min, variable depending on size of the dataset**

Raw UPE traces are organized in a structure variable and used as input for all the scripts.

1. Store UPE time trajectories as a structure variable named *UPEs.mat* dataset, with each field corresponding to a specific electrode type (i.e., Background, Occipital, and Temporal) or specific electrode type-task combinations (i.e., Bacground_Task1, Background_Task2, Occipital_Task1, Occipital_Task2, etc.).

***Note:*** For simplicity, examples are provided on electrode type only. Each field contains a table where rows represent time points and columns correspond to individual subjects. A variable of example is provided at Zenodo: https://doi.org/10.5281/zenodo.15830349. The variable *UPEs.mat* needs to be in the same folder as the scripts and will be used as input for all the scripts, unless stated otherwise.

***Note:*** If the raw trace requires filtering before analysis based on specific tasks, it is best to filter the entire trace first. Pre-filtering the entire trace helps maintain data integrity throughout the workflow. Filter types may differ for EEG and PMT data. Users should be aware that some filters may introduce time delays, which could impact workflow.

***Note:*** If the UPEs have been saved in an Excel file, use *OrganizeUPEsExcelFile.mat* script to create the *UPEs.mat* structure variable. The data in the Excel file needs to be structured with each sheet named for either the electrode or the electrode-task pair and within each sheet, columns represent different subjects, with the first row indicating subject ID. Rows correspond to time points.

***Note:*** Excel example file can be found in the folder (*Electrode-Task File.xlsx*).

### Hierarchical clustering

**Timing: 5–10 min, variable depending on size of the dataset**

Hierarchical clustering was performed on the raw UPE time traces to assess the temporal similarity across conditions/participants to identify recurring patterns (how the signals change over time).

2. Run *HierarchicalClusteringUPEs.m* script to obtain dendrograms (clustered traces) and heatmaps revealing patterns of temporal similarity in UPE traces:

>filename='UPEs.mat’;

>load (filename)

>numSubjects=20;

>names=fieldnames(UPEs);

>numPoints=size(UPEs.(names{1,1}),1);

>pos=1;

>numTraces=numSubjects∗size(names,1);

>data=NaN(numTraces,numPoints);

>for n=1:size(names,1)

> field=names{n,1};

> for i=1:numSubjects

> trace=UPEs.(field){:,i};

> data(pos,:)=trace';

> pos=pos+1;

> end

>end

>cg=clustergram(data,'Cluster', …, >'Column','Standardize','Row');

***Note:*** Parameters specifying the input data are 1) filename: the name of the structure variable. The structure variable UPEs contains fields corresponding to different electrode placements. For each field, a table stores the temporal trajectory of UPE values, with columns representing subjects and rows representing time points; 2) numSubjects: number of subjects analyzed; 3) 'Cluster': Dimension for data clustering. When set as ‘Column’, the function clusters along the columns of data (time) and reorders the rows (subject-electrode pair) accordingly. The dendrogram will be shown for the rows. In this way, the temporal trajectory is used to estimate the similarity between different pair of traces; 4) ‘Standardize’: Dimension for standardizing data values. When set as ‘Row’, the function standardizes each row (subject-electrode pair) to have mean 0 and std 1. This makes all traces comparable in scale before computing distances between columns. Emphasis is on comparing temporal patterns rather than absolute values.

***Note:*** Output is cg: Hierarchical cluster tree or dendrogram showing the grouping of time series based on their similarity.

***Note:*** Assumptions for this approach include Signal stationarity.

***Note:*** Pitfalls of this approach include: 1) Clustering may be driven by artifacts or noise; 2) Arbitrary selection of where to cut the dendrogram (see below); 3) The standardization of the signal removes amplitude information; 4) Highly nonlinear relationships are not captured.

***Note:*** The output is a dendrogram, where each branch represents a cluster, and the height of the branch is proportional to the similarity between data points. The decision of where to cut a dendrogram, namely how many clusters to define, depends on both the data and the goal of the analysis. A common approach is to look at the heights of the branches: larger gaps suggest more distinct clusters. You can also choose a fixed number of clusters (i.e., equivalent to the number of electrodes). Validation metrics like silhouette scores (*silhouette.m* function) and *evalclusters.m* function help guide the choice.

### Descriptive statistics

**Timing: 1–2 min, variable depending on size of the dataset**

Average UPE count and coefficient of variation (CV) are computed for all the traces.

3. Run *DescriptiveStatisticsUPEs.m* script to obtain average UPE count and CV (as a proxy of signal variability) for each trace:

>filename='UPEs.mat’;

>load (filename)

>numSubjects=20;

>names=fieldnames(UPEs);

>for n=1:size(names,1)

> field=names{n,1};

> for i=1:numSubjects

> trace=UPEs.(field){:,i};

> Average.(field)(i,1)=mean(trace);

> CV.(field)(i,1)=std(trace)/Average.(field)(i,1);

> end

>end

>filenameExcel1 = 'AverageResults.xlsx';

>filenameExcel2 = 'CVResults.xlsx';

>for i = 1:length(names)

> sheetName = names{i};

> data = Average.(sheetName);

> writematrix(data, filenameExcel1, 'Sheet', sheetName);

> data2 = CV.(sheetName);

> writematrix(data2, filenameExcel2, 'Sheet', sheetName);

>end

***Note:*** Parameters specifying the input data are 1) filename: the name of the structure variable. The structure variable UPEs contains fields corresponding to different electrode placements. For each field, a table stores the temporal trajectory of UPE values, with columns representing subjects and rows representing time points; 2) numSubjects: number of subjects analyzed.

***Note:*** Output: 1) Average: structure variable containing average values. Each field is a different electrode. For each field, values are stored as column, with each row corresponding to a different subject; 2) CV: structure variable containing average values. Each field is a different electrode. For each field, values are stored as column, with each row corresponding to a different subject; 3) Excel Files with average and CV values saved as Excel file. Each row is a different subject.

***Note:*** Coefficient of variation (CV) has been used as a proxy of signal variability. Alternatives include interquartile range (IQR), root mean square of successive differences and signal-to-noise ratio.

### Shannon entropy

**Timing: 2–5 min, variable depending on size of the dataset**

UPE entropy is computed for all the traces as a proxy of signal complexity/irregularity. It quantifies the unpredictability of the signal’s fluctuations over the recording time. High entropy suggests rich/flexible signal dynamic, whether low entropy may indicate increased regularity and loss of adaptive variability of the signal.

4. Run *ShannonEntropyUPEs.m* script to compute Shannon Entropy:

> filename='UPEs.mat';

>load (filename)

>numSubjects=20;

>%% Freedman Diaconis Rule

>names=fieldnames(UPEs);

>binWidth=0;

>for n=1:size(names,1)

> field=names{n,1};

> traces=UPEs.(field){:,:};

> Qb=iqr(traces);

> Minb=min(traces);

> Maxb=max(traces);

> Npoints=size(traces,1);

> binW=ceil((Maxb-Minb)./(2.∗Qb.∗Npoints.^∧^(-1/3)));

> binWidth=[binWidth binW];

>end

>binWidth(1)=[];

>Width=ceil(median(binWidth));

>for n=1:size(names,1)

> field=names{n,1};

> if n==1

> allData=UPEs.(field){:,:};

> else

>  allData=[allData;UPEs.(field){:,:}];

> end

>end

>binEdges = min(allData,[],'all'):Width:max(allData,[],'all');

> binEdges(end) = eps+binEdges(end);

>%% Shannon Entropy

>for n=1:size(names,1)

> field=names{n,1};

> for i=1:numSubjects

> trace=UPEs.(field){:,i};

> htot=histcounts(trace,binEdges);

> pTrace=htot/sum(htot);

> Entropy.(field)(i,1)=-sum(pTrace.∗log2(pTrace+eps));

> end

>end

>filenameExcel = 'Results_Step4_Entropy.xlsx';

>for i = 1:length(names)

> sheetName = names{i};

> data = Entropy.(sheetName);

> writematrix(data, filenameExcel, 'Sheet', sheetName);

>end

***Note:*** Parameters specifying the input data are 1) filename: the name of the structure variable. The structure variable UPEs contains fields corresponding to different electrode placements. For each field, a table stores the temporal trajectory of UPE values, with columns representing subjects and rows representing time points; 2) numSubjects: number of subjects analyzed.

***Note:*** Output: 1) Entropy: structure variable containing entropy values. Each field is a different electrode. For each field, values are stored as column, with each row corresponding to a different subject; 2) Excel File with entropy values saved as Excel file. Each row is a different subject.

***Note:*** It answers the question: What is the level of irregularity or complexity in the signal?

***Note:*** Assumptions for this approach include: 1) Stationarity of the signals is required. Common strategy to overcome this includes analyze shorter time windows; 2) The signal should possess sufficient dynamic range to allow meaningful discretization into bins; 3) The application of a common binning scheme (Freedman-Diaconis rule, see below) assumes that the overall distribution across signals is comparable.

***Note:*** Pitfalls of this approach include: 1) Shannon entropy is sensitive to noise. Noise can inflate entropy estimates and be mistaken for increased complexity; 2) Binning strategy influences the readouts; 3) Short time interval might yield unstable estimates. Tradeoff between window length and preservation of stationarity must be considered.

***Note:*** The Freedman-Diaconis rule is a statistical method used to determine the optimal bin width for constructing histograms. When computing entropy across multiple traces it becomes crucial to use a consistent and robust binning strategy. To compute the optimal bin width for each trace, the Freedman-Diaconis rule is applied to all traces and then the median value across all traces is used to define a unified bin width and compute a single bin edge list for all the traces. This is a practical approach that ensures a standardized and data-driven discretization method balancing individual variability with overall consistency. This is essential as it minimizes bias from arbitrary bin choices and improves reliability and comparability across subjects and conditions.

***Note:*** Shannon entropy is a commonly used measure of uncertainty due to its simplicity and interpretability.^9^ Common pitfalls include the sensitive to noise and binning choices. Alternatives include sample entropy, approximate entropy, and permutation entropy. These computations offer improved robustness for time series analysis, each with trade-offs in sensitivity and computation that needs to be factored by the researcher.

***Note:*** The analysis should be conducted separately for each task to avoid mixing segments from different tasks within the same trace.

### Frequency analysis—Short Fourier transformation

**Timing: 5–10 min, variable depending on size of the dataset**

Spectrogram (time-frequency representation) is computed using the short Fourier transformation (short FFT) approach.^10^ The spectrogram is a visual representation of the signal’s frequency content as it changes over time.

5. Run *ShortFFT_UPEs.m* script to compute the spectrogram:

**Figure 2:**
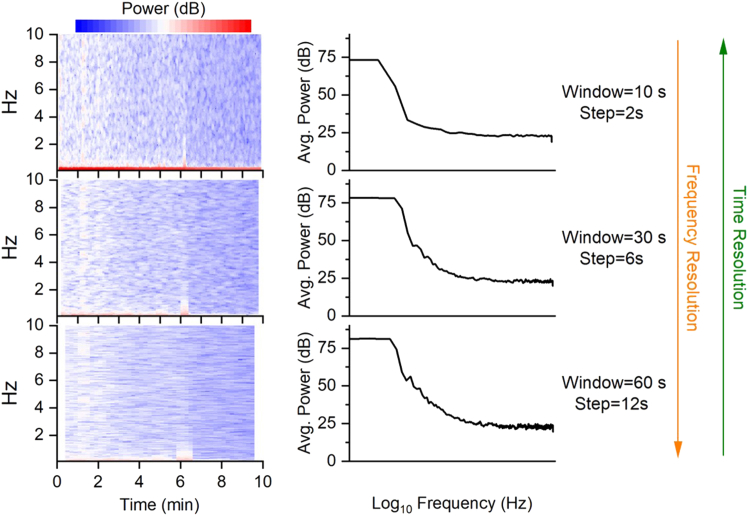
Time-Frequency trade off **Left**: Representative spectrograms with different time windows and steps (from smaller to larger), as indicated. **Right**: average power over frequencies for each spectrogram. Longer windows improve frequency resolution at the cost of time resolution. As evident from the graphs, improving the temporal resolution (top panel) comes at the cost of reduced frequency resolution, and vice versa.

>filename='UPEs.mat';

>load (filename)

>fs=20;

>timeWindow=20;

> overlapLength=0.75;

>numSubjects=20;

>window=timeWindow∗1000∗fs/1000;

>overlapLenght=round(overlap∗window);

>opt={'Window',hann(window),'OverlapLength',overlapLenght};

>names=fieldnames(UPEs);

>for n=1:size(names,1)

> field=names{n,1};

> for i=1:numSubjects

> trace=UPEs.(field){:,i};

> totalRecording=(size(trace,2)/(fs∗60));

> [s,f,t]=stft(trace,fs,opt{:});

> power=abs(s).ˆ2;

> PSD=10∗log10(abs(s).ˆ2);

> subfieldName=[field num2str(i)];

> Spectrogram.(subfieldName).power=PSD;

> Spectrogram.(subfieldName).frequency=f;

> Spectrogram.(subfieldName).time=t;

> end

>end

> save('Spectrogram',"Spectrogram")

***Note:*** Parameters specifying the input data are 1) filename: the name of the structure variable. The structure variable UPEs contains fields corresponding to different electrode placements. For each field, a table stores the temporal trajectory of UPE values, with columns representing subjects and rows representing time points; 2) fs: sampling frequency (in Hz); 3) timeWindow: length of the analysis window in seconds used to compute each FFT segment; 4) overlapLength : fraction of window overlaps between two consecutive windows, helping to reduce artifacts and improve time resolution; 5) numSubjects: number of subjects analyzed; 6) opt: an options structure specifying parameters for the spectral estimation (window type and overlap length).

***Note:*** Output: 1) Spectrogram: Variable structure containing fields named as the combination of the electrode and the subject number. For field contains a) power: nxm matrix, with each entry corresponding to power in Db (decibel). Rows (n) corresponding to frequency intervals, and columns (m) to time points; b) frequency: a nx1 column vector, with each entry corresponding to a frequency (Hz). c) time: a mx1 column vector, with each entry corresponding to a time (s).

***Note:*** Assumptions for this approach include: 1) The signal should not contain transient artifacts.

***Note:*** Pitfalls of this approach include: 1) Temporal and frequency tradeoff; 2) Spectral leakage (which is the artificial spreading of signal power across adjacent frequencies) might persist even with windowing.

***Note:*** The choice of the *timeWindow* is a tradeoff between time and frequency resolution. In fact, one cannot, at once, have a perfect time and frequency resolution; this is commonly referred to as the uncertainty principle in signal processing. A longer window improves frequency resolution (better separation of closely spaced frequencies) at the cost of reduced time resolution (it is harder to detect rapid changes). A shorter window enhances time resolution at the cost of frequency precision. The time window should be selected based on the characteristics of the signal: use longer windows for signals with stable, narrowband features, and shorter windows when capturing transient events or fast-changing dynamics. A common starting point is to select several *timeWindows* sizes, plot the spectrograms and visually inspect the results. Examples are shown in Figure 2.

***Note:*** The choice of the *overlapLength* is a tradeoff between time and frequency resolution. A higher overlap (e.g., 50%–75% of the window length) reduces artifacts caused by windowing and provides smoother time-frequency representations, making it easier to track fast-changing frequency components. Overall, more overlap is indicated for non-stationary signals, while less overlap is indicated for slower changes.

***Note:*** The window function (i.e., Hann) is used to reduce spectral leakage. Since the short FFT assumes the signal is periodic within each segment, abrupt edges in the signal can create artifacts in the spectrum —known as spectral leakage. Applying a window function tapers the signal smoothly at the edges, minimizing these discontinuities. This leads to a more accurate representation of the signal’s true frequency content.

***Note:*** The analysis should be conducted separately for each task to avoid mixing segments from different tasks within the same trace.

***Note:*** Short FFT has limitations: it offers only moderate spectral resolution, suffers from a time–frequency resolution trade-off, and exhibits high variance and spectral leakage unless averaged. See troubleshooting 1 for potential solution.

6. Run *Plot_spectrogram_UPEs.m* to plot spectrograms of selected traces:

>filename='Spectrogram.mat';

>load (filename)

>fs=20;

>timeWindow=20;

>names=fieldnames(Spectrogram);

>[indx,∼] = listdlg('ListString',names);

>howMany=size(indx,2);

>for i=1:howMany

> figure(i)

> isPos=indx(1,i);

> name=names{isPos,1};

> trace=Spectrogram.(name).power;

> f=Spectrogram.(name).frequency;

> t=Spectrogram.(name).time;

> minFreq=(1/timeWindow)∗2;

> [∼, index] = min(abs(f-minFreq));

> image([0 t(end,1)],[minFreq,fs/2],…,

> trace(index:end,:),'CDataMapping','scaled');

> colormap('jet')

> a=colorbar;

> a.Label.String = 'Power (db)';

> title(name)

> xlabel('Time (s)')

> ylabel('Frequency (Hz)')

>end

***Note:*** Parameters specifying the input data are 1) filename: the name of the spectrogram structure variable saved used the script in Step5 (*ShortFFT_UPEs.m*); 2) fs: sampling frequency (in Hz); 3) timeWindow: length of the analysis window in seconds used to compute each FFT segment.

### DFA

**Timing: 15–20 min, variable depending on size of the dataset**

Many signals are not random, rather they exhibit a degree of temporal organization, which means that past states of a system (reflected in the values of the signal) influence its future behavior. Detrend fluctuation analysis (DFA) scaling exponent is computed as a proxy of long-range temporal correlations.^11^^,^^12^ In simple terms, DFA scaling exponent informs us about the signal’s “memory”: whether what the signal is doing now is related to what it was doing earlier, even across long time intervals. DFA scaling exponents can be lower than 0.5 (anti-correlated dynamics; anti-persistence behavior), equal to 0.5 (uncorrelated; white noise-like behavior), between 0.5 and 1 (long-range positive correlations; persistence behavior) and greater than 1 (strongly correlated or non-stationary behavior, often reflecting oscillatory or non-scaling properties).

7. Run *ComputeDFA_upe_singleWindow.mat* to select the fitting interval (FitInterval), calculation interval (CalcInterval) and filter design properties to compute the DFA exponent. Use white noise traces to confirm the validity of the pipeline. Once the parameters are determined, run *ComputeDFA_UPE_Loop.mat* to run the DFA for all the UPE traces.

>filename='UPEs.mat';

>load (filename)

>fields=fieldnames(UPEs);

>hp=0.1;

>lp=1;

>fs=20;

>filter_order=50/hp;

>FitInterval=[80 150];

>CalcInterval=[20 200];

>overlap=0.8;

>Wn = [hp/(fs/2), lp/(fs/2)];

>designedFilter = fir1(filter_order, Wn, 'bandpass', hann(filter_order + 1),'scale');

>for j=1:size(fields,1)

> Electrode=fields{j,1};

> for i=1:20

> Signal=UPEs.(Electrode){:,i};

> Signal = Signal - mean(Signal);

> Data_filtered = filter(designedFilter,1,Signal);

>[AmplitudeEnvelope,AmplitudeEnvelopeInfo]=nbt_GetAmplitudeEnvelope_v2(Data_filtered, hp, lp);

>[DFAobject,DFA_exp]=nbt_doDFA(AmplitudeEnvelope,AmplitudeEnvelopeInfo, FitInterval, CalcInterval, overlap, 0, 10,fs);

> Exponent(i,j)=DFA_exp;

> fprintf('%.3f, %s - trace %i\n',DFA_exp,Electrode,i)

> end

>end

>DFAValues=array2table(Exponent,'VariableNames',fields);

>InputValues=table(hp, lp, fs, filter_order, FitInterval, CalcInterval, overlap);

>writetable(DFAValues, 'DFA Scaling Exponent.xlsx','Sheet','DFA exp');

>writetable(InputValues, 'DFA Scaling Exponent.xlsx','Sheet','Input');

***Note:*** Parameters specifying the input data are 1) filename: the name of the structure variable. The structure variable UPEs contains fields corresponding to different electrode placements. For each field, a table stores the temporal trajectory of UPE values, with columns representing subjects and rows representing time points; 2) Electrode: name of the electrode to use; 3) hp: high-pass filter; 4) lp: low-pass filter; 5)fs: sampling frequency; 6) filter_order: filter order for the bandpass filter; 7) FitInterval: smallest and largest time scale to include in power-law fit (in seconds); 8) CalcInterval: minimum and maximum time-window size computed (in seconds); 9) overlap: fraction of overlap between consecutive windows; 10) desingedFilter: window-based FIR filter design input; 11) numberWhiteNoise: number of white noise traces to generate.

***Note:*** Output for *ComputeDFA_UPE_singleWindow.m:* 1) DFA White Noise (printed in the command window) - for the given set of input, this value should be as close as possible to 0.5 (uncorrelated); 2) DFA value of Trace XX (printed in the command window) - for the given set of input, this is the DFA scaling exponent value for the trace selected (XX); 3) Plot showing the fit interval (red circles, red line) and relative DFA scaling exponent.

***Note:*** Output for *ComputeDFA_UPE_Loop.m:* 1) DFAValues: table containing for each electrode, the DFA scaling exponent values (each row is a different subject); 2) DFAValues and input parameters saved as Excel file.

***Note:*** It answers the question: Does the signal have long-range temporal correlations (i.e., memory)?

***Note:*** Assumptions for this approach include: 1) The data should be long enough to allow reliable estimation across multiple time scales; 2) The signal should be approximately stationary; 3) Filtering must preserve the temporal correlation of interest.

***Note:*** Pitfalls of this approach include: 1) Aggressive or poorly designed filters can introduce temporal correlations; 2) DFA scaling exponential can arise from different mechanisms, thus limiting mechanistic conclusions without additional validation.

***Note:*** DFA is sensitive to the filtering procedure, particularly to the value of the filter_order. When evaluating the filter using *fvtool.mat* function, several features should be examined to assess its quality. First, the passband must be flat with no ripples, guaranteeing minimal distortion of the signal of interest. Second, the transition bands should roll off smoothly without being too steep, which helps avoid ringing artifacts. Third, no oscillations or ripples should be evident near the edges, which could indicate poor time-domain behavior. Finally, the phase response should be linear, preserving the shape of the signal over time. The Hann window helps reduce spectral leakage and ensures smooth transitions between passband and stopband, minimizing ripples and ringing in the time domain. The ‘scale’ option normalizes the filter gain in the passband, ensuring that the signal amplitude is preserved. Together, these choices result in a filter that cleanly isolates the 0.1–1 Hz range with minimal distortion, making it suitable for analyzing slow physiological or neural fluctuations. Troubleshooting 2 section showed examples of filters.

***Note:*** Subtracting the mean before DFA ensures the signal is centered at zero. This prevents a false trend from appearing during integration, which could distort the results. Centering helps DFA accurately measure the true fluctuations and long-range correlations in the data.

***Note:*** FitInterval is the range of time scales over which the scaling exponent α is estimated and needs to be chosen as a subset of the full fluctuation plot where the power-law behavior is most reliable and linear. FitInterval needs to be within the CalcInterval window, which is the full range of time scales considered in the DFA analysis, ensuring that the fit is based only on meaningful, stable fluctuations and not influenced by noise or edge effects at very short or long-time scales.

***Note:*** It is important to test the same filter, FitInterval, and CalcInterval settings on white noise traces (n=1000) before applying the pipeline to the signal. Since white noise has no intrinsic correlations (exponent close to 0.5) it serves as a baseline for comparison. By analyzing white noise with the same pipeline, we verify that the method does not falsely detect long-range correlations where none exist. If the average DFA scaling exponent is close to 0.5 — its theoretical value — it confirms that the analysis setup is working correctly and any deviation in the UPE signals likely reflects true biological correlations rather than artifacts of the method. We used a cutoff of DFA scaling exponent of 0.5±0.03. For example, while the FitInterval 35-80 s is linear, the average DFA scaling exponent of the white noise traces is 0.54. Accordingly, the range 80-150 s (scaling exponent of white noise traces=0.51) has been used for the current analysis. **Troubleshooting 3** for white noise and linear interval provided.

### Correlation analysis

**Timing: 5–10 min, variable depending on size of the dataset**

Pairwise correlation analysis between electrodes (Background, Occipital and Temporal) is computed as a proxy of temporal synchronicity.

8. Run *ComputeCorrelationUPEs.mat* to compute pairwise correlation analysis.

***Note:*** Parameters specifying the input data are 1) filename: the name of the structure variable. The structure variable UPEs contains fields corresponding to different electrode placements. For each field, a table stores the temporal trajectory of UPE values, with columns representing subjects and rows representing time points; 2) ResempleFactor: resampling factors. If 1, no resampling; 3) cutoff: threshold for eliminating weak edges; 4) fs: sampling frequency (Hz); 5) TimeWindow: length of the time window (in seconds); 6) Overlap: proportion of overlap between consecutive time windows (as a fraction of window length); 7) numSubjects: number of subjects.

***Note:*** Output: 1) NetworksCorrelation: structure variable. For each subject, each field is the network (matrix of all pairwise comparisons) at each time window; 2) NetworkEdgeTrajectories: structure variable. For each subject, each field is a combination of electrodes (i.e., BaOc is Background and Occipital. Ba - Background; Oc - Occipital; Te - Temporal). For each field, the correlation values at each time window are stored; 3) Corr: structure variable. Each field is a combination of electrodes (i.e., BaOc is Background and Occipital. Ba - Background; Oc - Occipital; Te - Temporal). For each field, the median correlation values across all time windows are stored. Each row is a different subject.

***Note:*** Assumptions for this approach include 1) Signal free from artifacts; 2) Signal should be stationary; 3) Approximately normally distributed values if Pearsons’s correlation used.

***Note:*** Pitfalls of this approach include 1) Pairwise correlation does not distinguish between direct/indirect connections; 2) Correlation is sensitive to volume conduction and third-party effects; 3) Correlation is sensitive to outliers; 4) Correlation assumes linearity of interaction; nonlinear relationship is potentially missed.

***Note:*** The analysis should be conducted separately for each task to avoid mixing segments from different tasks within the same trace.

***Note:*** Selecting an appropriate cutoff is essential to ensure that the resulting network reflects meaningful interactions while minimizing noise and spurious connections. A well-justified cutoff preserves key structural properties, enhances interpretability, and ensures computational efficiency, allowing for more accurate analysis and comparison across networks.

***Note:*** While the proposed analysis primarily focuses on median values across time (for the sake of conceptual clarity and interpretability) this approach involves compressing time. As a result, we effectively treat what is inherently a dynamic network as if it were static. This simplification may obscure potentially meaningful temporal variations and limit the ability to capture time-sensitive changes in the network. To mitigate this limitation, we also provide the full set of time-varying metrics and networks at each time point. These allow for a more nuanced, temporally sensitive analysis that can reveal dynamic patterns and dependencies that would otherwise remain hidden in the aggregated view. Researchers are therefore encouraged to consider both perspectives: the simplified median-based representation for high-level interpretation, and the time-resolved data for more detailed, dynamic network analyses. For further details on temporal network analysis, see.^13^

***Note:*** To mitigate the effects of volume conduction and a third-party variable, Pearson's correlation analysis can be replaced with partial correlation. Partial correlation (*partialcorr.mat*) returns the sample linear correlation between a pair of signals controlling for other signals. The rationale is that the influence of common sources or third-party effects is statistically reduced, allowing for the detection of more direct relations.

### Coherence analysis

**Timing: 5–10 min, variable depending on size of the dataset**

Coherence measures how consistently two signals oscillate together at a given frequency band. Median pairwise coherence analysis across all frequencies and between electrodes (Background, Occipital, and Temporal) is computed as a proxy of frequency synchronicity. If two signals show high coherence, it suggests that they are coordinating activity at that frequency (or frequency band).

9. Run *ComputeCoherenceUPEs.mat* to compute pairwise correlation analysis.

***Note:*** Parameters specifying the input data are 1) filename: the name of the structure variable. The structure variable UPEs contains fields corresponding to different electrode placements. For each field, a table stores the temporal trajectory of UPE values, with columns representing subjects and rows representing time points; 2) ResampleFactor: resampling factors. If 1, no resampling; 3) cutoff: threshold for eliminating weak edges; 4) fs: sampling frequency (Hz); 5) TimeWindow: length of the time window (in seconds); 6) Overlap: proportion of overlap between consecutive time windows (as a fraction of window length); 7) numSubjects: number of subjects.

***Note:*** Output: 1) NetworksCoherence: structure variable. For each subject, each field is the network at each time window; 2) NetworkEdgeTrajectories: structure variable. For each subject, each field is a combination of electrodes (i.e., BaOc is Background and Occipital. Ba - Background; Oc - Occipital; Te - Temporal). For each field, the coherence values at each time window are stored; 3) Coherence: structure variable. Each field is a combination of electrodes (i.e., BaOc is Background and Occipital. Ba - Background; Oc - Occipital; Te - Temporal). For each field, the median coherence values across all time windows are stored. Each row is a different subject.

***Note:*** It answers the question: How consistently two signals oscillate together at specific frequencies?

***Note:*** Assumptions for this approach include: 1) The signal is weakly non-stationary within each time window; 2) The sampling frequency is sufficiently high to capture the frequency bands of interest according to the Nyquist criterion (i.e., at least twice/three times the highest frequency analyzed); 3) The length of the *TimeWindow* provides sufficient data points for robust spectral estimation (typically requiring windows of at least 1–2 seconds depending on the lowest frequency of interest).

***Note:*** Pitfalls of this approach include: 1) Coherence is sensitive to volume conduction and common reference effects; 2) Pairwise coherence cannot distinguish direct from indirect connections.

***Note:*** The analysis should be conducted separately for each task to avoid mixing segments from different tasks within the same trace.

***Note:*** Selecting an appropriate cutoff is essential to ensure that the resulting network reflects meaningful interactions while minimizing noise and spurious connections. A well-justified cutoff preserves key structural properties, enhances interpretability, and ensures computational efficiency, allowing for more accurate analysis and comparison across networks.

***Note:*** While the proposed analysis primarily focuses on median values across time (for the sake of conceptual clarity and interpretability) this approach involves compressing time. As a result, we effectively treat what is inherently a dynamic network as if it were static. This simplification may obscure potentially meaningful temporal variations and limit the ability to capture time-sensitive changes in the network. To mitigate this limitation, we also provide the full set of time-varying metrics and networks at each time point. These allow for a more nuanced, temporally sensitive analysis that can reveal dynamic patterns and dependencies that would otherwise remain hidden in the aggregated view. Researchers are therefore encouraged to consider both perspectives: the simplified median-based representation for high-level interpretation, and the time-resolved data for more detailed, dynamic network analyses. For further details on temporal network analysis, see.^13^

***Note:*** To mitigate the effects of volume conduction, third-party variable, and provide directed results (i.e., differentiate between the coherence of Signal A to B and vice versa), partial directed coherence should be investigated. Partial directed coherence returns frequency-specific measures identifying the direction of influence between two signals. It allows to understand to which degree one signal drives another at specific frequencies, while removing the effects of shared sources or indirect connections.

### Granger causality

**Timing: 15–20 min, variable depending on size of the dataset**

Granger causality measures whether knowing the past of Signal A improves the prediction of the Signal B’s future beyond what you could predict from Singal B’s own past alone. Granger causality between pairwise electrodes (Background, Occipital, and Temporal) is computed as a proxy of directed functional connectivity; the remaining trace(s) is used as a conditional variable. This is important because it accounts for indirect influences and isolates the direct interaction between the two regions being tested. It is useful for identifying which signal “leads” and which “follows”.

10. Run *ComputeGrangerCausalityUPEs.mat* to compute pairwise correlation analysis.

***Note:*** Parameters specifying the input data are 1) filename: the name of the structure variable. The structure variable UPEs contains fields corresponding to different electrode placements. For each field, a table stores the temporal trajectory of UPE values, with columns representing subjects and rows representing time points; 2) ResampleFactor: resampling factors. If 1, no resampling; 3) cutoff: threshold for eliminating weak edges. Since Granger Causality computation involves a statistical test, edges corresponding to non-significant pairwise interactions (p > 0.05) are set to zero; 4) fs: sampling frequency (Hz); 5) TimeWindow: length of the time window (in seconds); 6) Overlap: proportion of overlap between consecutive time windows (as a fraction of window length); 7)numSubjects: number of subjects.

***Note:*** Output: 1) NetworksGrangerCausality: structure variable. For each subject, each field is the network at each time window; 2) NetworkEdgeTrajectories: structure variable. For each subject, each field is a combination of electrodes (i.e., BaOc is Background and Occipital. Ba - Background; Oc - Occipital; Te - Temporal). For each field, the Test statistic values at each time window are stored; 3) GrangerCausality: structure variable. Each field is a combination of electrodes (i.e., BaOc is Background and Occipital. Ba - Background; Oc - Occipital; Te - Temporal). For each field, the median test statistic values across all time windows are stored. Each row is a different subject.

***Note:*** It answers the question: Does one signal help predict the future of another?

***Note:*** Assumption for this approach include: 1) The signal should be stationary; 2) The signal should be sufficiently long relative to the model order (how many points in the past are used to make the prediction).

***Note:*** Pitfalls of this approach include: 1) Susceptibility to false positives; 2) Misinterpretation of Granger Causality with true mechanistic causation; 3) Sensitivity to the model order: in the present pipeline, the default model order (number of lags equal to 1; namely, one point in the past is used for the analysis) is used.

***Note:*** The analysis should be conducted separately for each task to avoid mixing segments from different tasks within the same trace.

***Note:*** Selecting an appropriate cutoff is essential to ensure that the resulting network reflects meaningful interactions while minimizing noise and spurious connections. A well-justified cutoff preserves key structural properties, enhances interpretability, and ensures computational efficiency, allowing for more accurate analysis and comparison across networks.

***Note:*** Considering the susceptibility to false positives, the Granger Causality pipeline can be implemented with phase-randomized surrogates. Phase-randomized surrogates are used to generate a null distribution. This method randomizes the phase of the Fourier transform for each channel independently, preserving the power spectrum and autocorrelation while destroying any true temporal dependencies. By computing Granger causality on multiple surrogate datasets, a distribution of values under the null hypothesis (no real causal influence) is constructed. The empirical p-value is then determined by comparing the original Granger causality value to this null distribution, allowing rejection of false positives arising from noise, overfitting, or model artifacts. Sufficient surrogates (n>250) are required to ensure reliable significance testing. If the original value passes this test, it can be then tested using Time-reversed Granger causality (TRGC). TRGC is used to distinguish directed influences from spurious connectivity (volume conduction or third-party effect). This approach revers the time axis of the signals, computing Granger causality on both the original (GC_fwd_) and time-reversed (GC_rev_) data and comparing them. The TRGC index is calculated as:

$$TRGC\;index=\frac{{GC}_{fwd}-{GC}_{rev}}{{GC}_{fwd}+{GC}_{rev}}$$

If index greater than 0.3, then the GC value is asymmetric, and likely reflects more robust causality.

***Note:*** While the proposed analysis primarily focuses on median values across time (for the sake of conceptual clarity and interpretability) this approach involves compressing time. As a result, we effectively treat what is inherently a dynamic network as if it were static. This simplification may obscure potentially meaningful temporal variations and limit the ability to capture time-sensitive changes in the network. To mitigate this limitation, we also provide the full set of time-varying metrics and networks at each time point. These allow for a more nuanced, temporally sensitive analysis that can reveal dynamic patterns and dependencies that would otherwise remain hidden in the aggregated view. Researchers are therefore encouraged to consider both perspectives: the simplified median-based representation for high-level interpretation, and the time-resolved data for more detailed, dynamic network analyses. For further details on temporal network analysis, see.^13^

***Note:*** It is important to stress that Granger causality does not prove by itself true causation, rather it reveals directional influence by quantifying predictive power and not mechanistic causation. If Signal A Granger Cause Signal B, we can say that Signal A helps predict Signal B.

### Mutual information

**Timing: 5–10 min, variable depending on size of the dataset**

Mutual Information between pairwise electrodes (Background, Occipital, and Temporal) is computed as a proxy of shared information between traces. This quantifies how much knowing the state of one electrode reduces uncertainty about the state of another, reflecting functional connectivity. Unlike coherence or linear correlation, mutual information captures any statistical dependency, whether linear (output is directly proportional to the input) or non-linear (output is not directly proportional to the input; interactions, threshold, feedback, state-dependent dynamics might cause this behavior). Considering that many biological systems rely on nonlinear dynamics, it is important to analyze the signals with a tool robust to nonlinear effects.

11. Run *ComputeMutualInformationUPEs.mat* to compute pairwise correlation analysis.

***Note:*** Parameters specifying the input data are 1) filename: the name of the structure variable. The structure variable UPEs contains fields corresponding to different electrode placements. For each field, a table stores the temporal trajectory of UPE values, with columns representing subjects and rows representing time points; 2) ResampleFactor: resampling factors. If 1, no resampling; 3) cutoff: threshold for eliminating weak edges; 4) fs: sampling frequency (Hz); 5) TimeWindow: length of the time window (in seconds); 6) Overlap: proportion of overlap between consecutive time windows (as a fraction of window length); 7) numSubjects: number of subjects; 8) K: k-th nearest neighbor.

***Note:*** Output: 1) NetworksMutualInformation: structure variable. For each subject, each field is the network at each time window; 2) NetworkEdgeTrajectories: structure variable. For each subject, each field is a combination of electrodes (i.e., BaOc is Background and Occipital. Ba - Background; Oc - Occipital; Te - Temporal). For each field, the mutual information values at each time window are stored; 3) MutualInformation: structure variable. Each field is a combination of electrodes (i.e., BaOc is Background and Occipital. Ba - Background; Oc - Occipital; Te - Temporal). For each field, the median mutual information values across all time windows are stored. Each row is a different subject.

***Note:*** It answers the question: How much does knowing the state of Signal A reduce the uncertainty — i.e., how much better can we predict or understand Signal B just by observing Signal A — regardless of whether their relationship is linear or nonlinear?

***Note:*** Assumption for this approach include: 1) The signal should be stationary.

***Note:*** Pitfalls of this approach include: 1) Sensitive to the choice of the k-nearest neighbor; 2) Approach does not distinguish directionality.

***Note:*** The analysis should be conducted separately for each task to avoid mixing segments from different tasks within the same trace.

***Note:*** Selecting an appropriate cutoff is essential to ensure that the resulting network reflects meaningful interactions while minimizing noise and spurious connections. A well-justified cutoff preserves key structural properties, enhances interpretability, and ensures computational efficiency, allowing for more accurate analysis and comparison across networks.

***Note:*** While the proposed analysis primarily focuses on median values across time (for the sake of conceptual clarity and interpretability) this approach involves compressing time. As a result, we effectively treat what is inherently a dynamic network as if it were static. This simplification may obscure potentially meaningful temporal variations and limit the ability to capture time-sensitive changes in the network. To mitigate this limitation, we also provide the full set of time-varying metrics and networks at each time point. These allow for a more nuanced, temporally sensitive analysis that can reveal dynamic patterns and dependencies that would otherwise remain hidden in the aggregated view. Researchers are therefore encouraged to consider both perspectives: the simplified median-based representation for high-level interpretation, and the time-resolved data for more detailed, dynamic network analyses. For further details on temporal network analysis, see.^13^

## Expected outcomes

**Figure 3:**
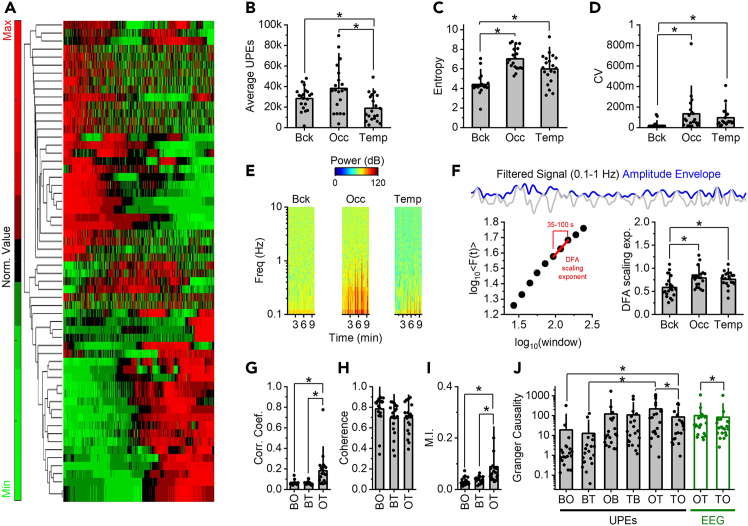
Brain UPE signals can be distinguished from background UPE signals (A–D) (A) Representative heatmap and dendrogram. Average (bar) and single values (circles) of (B) count, (C) coefficient of variation (CV) and (D) entropy. (E) Representative spectrograms. (F–J) (F) Representative filtered signal (gray) and amplitude envelope (blue). Amplitude envelope was then used for computing the DFA scaling exponent. Bar graph and single points of DFA scaling exponents are shown. Average and single values of (G) correlation coefficient, (H) coherence, (I) mutual information and (J) Granger causality of pairwise electrode signals. For Granger causality, the order matters (BO implies a directional value from background to occipital, whether OB a directional value from occipital to background. ∗ p < 0.05. See statistic section for details. Background: Bck, B; Occipital: Occ, O; Temporal: Temp, T.

Example results are provided in Figure 3. The hierarchical clustering (panel A) shown three major dynamics: signals that generally increased over time, signals that decreased, and signals that showed more irregular fluctuations. These dynamics reflect how the intensity of UPE signals evolves across time in different brain and background regions. Descriptive statistics (Panels B–D) showed that the average UPE signal intensity (mean counts) was not useful in distinguishing brain electrodes (occipital and temporal) from background electrodes. However, both entropy and coefficient of variation (CV)—which represent the diversity and variability of the signal, respectively—were significantly higher in brain regions. This suggests that UPEs from brains are more complex and varied than background signals, even if their average count is similar. Spectral analysis (Panel E) demonstrated that UPE signals from brain electrodes had greater power in the very low frequency range (<1 Hz). This indicates the presence of slow, gradual changes in UPE signal intensity in the brain, which were absent in the background signals. The detrended fluctuation analysis (DFA) (Panel F) provided a measure of temporal correlation—how strongly a signal’s past values predict its future values. The brain UPE signals showed higher DFA exponents, particularly in the 35–100 second range, indicating more persistent, structured fluctuations over time. Interestingly, even the background signals had a DFA exponent different from 0.5, meaning they were not completely random (white noise) and may still carry some structured, low-level signal. Pairwise correlation (Panel G) and mutual information (Panel I) showed that the brain UPE signals were more temporally coordinated, and they shared more informational content with each other than with background signals. These results point to stronger temporal dynamics (correlation) and functional connectivity (mutual information) within brain regions.

Coherence analysis (Panel H), which tests for frequency-specific synchronization between signals, did not reveal significant coherence within or between brain and background electrodes. This suggests that the UPE signals are not strongly coupled at specific frequencies, unlike traditional EEG oscillations. Finally, Granger Causality analysis (Panel J), which estimates directional influence between signals, indicated that the background signal did not causally affect the brain UPE signals. However, occipital and temporal brain regions influenced each other, with the occipital region more strongly driving the temporal region. This pattern is consistent with known brain activity flow between these regions (e.g., from O1 to T4 in EEG recordings simultaneously with UPE measures from the same subjects). Interestingly, both occipital and temporal regions also influenced the background signal, suggesting a possible leakage of brain-derived UPE signals into the surrounding environment, albeit in a degraded form. This interpretation is supported by the fact that background signals deviate from white noise, as seen in the DFA results. These networks approaches compressed temporal dynamics, effectively treating a dynamic network as static. This may obscure time-sensitive variations, particularly in cases with tasks. To address this limitation, future studies will include temporal network analysis, with a focus also on task-dependent changes.

## Quantification and statistical analysis

The data reported in Casey et al.^1^ was collected from 20 subjects, with three PMTs (background, occipital and temporal) measured simultaneously per subject. Each subject performed five tasks (eyes open pre music, eyes closed pre music, music, eyes closed post music and eyes open post music). Each dependent variable (i.e., CV, entropy, etc.) was a continuous score computed for each subject under each task and electrode combination, generating 15 observations per subject (3 electrodes × 5 tasks). This created a within-subjects repeated measures design, where each participant served as their own control across all experimental conditions. The advantages of the repeated measures design include accountability for individual differences, reduction of sample size and assessment of interacting effects. For each the metric reported in the expected outcomes figure, we can test several hypotheses.

To analyze how the score varied by electrode placement and task type, we used a linear mixed-effects model. Run *MixedEffectModels.R* to perform statistic:

># Linear Mixed-Effects Model Analysis Script

># 1. Load libraries

>library(readxl)

>library(lme4)

>library(lmerTest)

>library(tidyverse)

>library(car)

>library(emmeans)

># 2. Import the dataset

>file_path <- " "

>dataset <- read_excel(file_path)

>dataset$Subject <- as.factor(dataset$Subject)

>dataset$Electrode <- as.factor(dataset$Electrode)

>dataset$Task <- as.factor(dataset$Task)

># 3. Fit the mixed-effects model

>model <- lmer(Entropy ∼ Electrode ∗ Task + (1 | Subject) + (1 | >Subject:Task), data = dataset)

># 4. Check for singular fit

>if (isSingular(model)) {

> stop("Model is singular — random effects structure may be too >complex. Consider simplifying the model (e.g., removing random >slopes or interactions).")

>}

># 5. Display model summary

>cat("\nModel Summary:\n")

>print(summary(model))

># 6. Compute and display Type III ANOVA

>cat("\nType III ANOVA Results (Satterthwaite df):\n")

>anova_result <- Anova(model, type = "III")

>print(anova_result)

># 7. Post-hoc pairwise comparisons

>cat("\nPost-hoc pairwise comparisons (Bonferroni correction) >for Electrode:\n")

># Estimated marginal means

>emm_electrode <- emmeans(model, ∼ Electrode|Task)

>posthoc_results <- contrast(emm_electrode, method = >"pairwise", >adjust = "bonferroni", infer = c(TRUE, TRUE))

> sigma_resid <- sigma(model)

> posthoc_with_d <- summary(posthoc_results)

>mutate(`Cohen's d` = estimate / sigma_resid, `Cohen's d` = round(`Cohen's d`, 4), p.value = round(p.value, 4), estimate = round(estimate, 4), SE = round(SE, 4), t.ratio = round(t.ratio, 3), lower.CL = round(lower.CL, 4), upper.CL = round(upper.CL, 4))

>select(Task, contrast, estimate, SE, lower.CL, upper.CL, >t.ratio, p.value, `Cohen's d`)

>print(posthoc_with_d)

***Note:*** Parameters specifying the input data are 1) file_path: Path to the Excel file containing the dataset used for statistical analysis; 2) formula: A formula specifying the fixed and random effects structure of the linear mixed-effects model. To be indicated in the lmer function; 3) post-hoc correction: Type of post-hoc correction applied to adjust for multiple comparisons. Examples include Bonferroni, Tukey, Holm, or False Discovery Rate (FDR); 4) post-hoc comparison: type of post hoc comparisons to be indicated in the contrast function. Examples include Electrode, where pairwise comparisons are made between all levels of Electrode, averaged across Tasks; Electrode|Task, where pairwise comparisons between Electrodes are conducted separately for each Task; Task|Electrode, where pairwise comparisons between Tasks are conducted separately for each Electrode.

***Note:*** Output includes Analysis of Variance Table and post-hoc pairwise comparison including estimate, SE, lower and upper confidence intervals, t ratios, p-Values and Cohen’s effect sizes.

***Note:*** Mixed-effects models are more flexible and powerful than traditional repeated-measures ANOVA.^14^ They do not require strict assumptions like sphericity and can account for individual differences by including random effects. The proposed experimental design was a fully crossed design, where all levels of one independent factor (Task) are present within all levels of the other independent factor (Electrode), for each subject.

***Note:*** To appropriately model the experimental design structure, we considered several random effects, which allow the model to capture variability across individuals and within specific combinations of factors such as subject-task or subject-electrode. A random intercept allows the model to account for natural differences between subjects. A random slope allows the model to capture how effects (i.e., tasks or electrodes) vary across subjects. Questions include: 1) Do subjects differ in their average level of the measured dependent variable across all tasks and electrodes? A basic random intercept for each subject, (1 | Subject), accounts for baseline differences between individuals. This is a proxy of how much each subject’s overall performance differs from the average, regardless of task or electrode. 2) Does each subject have a different baseline level of measured dependent variable depending on where the electrode is placed? Does this pattern vary from one person to another? A random intercept for subject-by-electrode, (1| Subject:Electrode), allows for individual-specific baseline shifts depending on electrode placement regardless of the task. This is a proxy of how much a subject’s overall response differs based on where the electrode is placed, independent of what task they are doing. 3) Does each subject respond differently to each task regardless of where we measure the signal? A random intercept (1| Subject:Task), allows for individual-specific baseline shifts depending on task regardless of electrode placement. This is a proxy of how much a subject’s overall response differs based on task, independent of where the electrode is placed.

***Note:*** A random slope for Task within Subject, (Task | Subject) allows to assess whether individuals responded differently to the same set of tasks regardless of electrode placement (Question: Does the effect of the task on the dependent variable differ between subjects?). Likewise, a random slope for Electrode within Subject, (Electrode | Subject), allows to evaluate whether the impact of electrode placement varies across subjects regardless of task (Question: Does the effect of electrode placement differ between subjects?). A random factor that allows task to vary by Subject:Electrode , (Task | Subject:Electrode), reflects how task effects may be electrode-specific for each individual (Question: Does the effect of the task on the dependent variable differ across electrodes and vary from subject to subject?). Similarly, allowing the effect of Electrode to vary by Subject:Task, (Electrode | Subject:Task), allows to test whether electrode sensitivity changed depending on the task being performed (Question: Does the effect of electrode location on the dependent variable depend on which task the subject is performing?).

See troubleshooting 4 for potential solution on the decision of the model formula.

***Note:*** In mixed-effects models, the denominator degrees of freedom (ddf) reflect how much information is available to estimate the uncertainty of a fixed effect. Unlike in traditional repeated-measures ANOVA, where ddf are based only on the number of subjects, mixed models account for the fact that each subject contributes multiple observations (e.g., across tasks and electrodes). Advanced methods like Satterthwaite’s approximation use this richer data structure to compute ddf that can be much larger than the number of subjects, while still properly accounting for dependencies in the data.

## Limitations

One of the main limitations of the protocol is its incompatibility with neurocognitive tasks that involve the use of visual stimuli. Any extraneous source of light will significantly elevate dark counts in the recording chamber. Of course, any visually relevant stimuli would need to be directed toward the eyes – and by extension, the head – thus, interfering with PMT measurements. Because UPEs are many orders of magnitude less intense than any light source that might be used to display a face, visual oddball, maze or any number of stimuli to activate visual pathways, experimentalists will need to rely on other sensory modalities. While the use of virtual reality headsets or similar technologies could potentially be sealed off to prevent increased dark counts, there is evidence to suggest that light can travel directly through the brain and skull,^15^^,^^16^ which introduces a potential confound while measuring brain-based UPEs. There is also evidence to suggest that axons can serve as transmissive lightguides,^7^^,^^17^ complicating the interpretation of any PMT recordings obtained while using visual stimuli. In the present recording setup, we did not perfectly synchronize UPE and EEG measurements; proper synchronization will allow to perform robust EEG-UPE signal analysis at millisecond and sub-millisecond scales.

## Troubleshooting

### Problem 1

The Short-Time Fourier Transform (short FFT) (see Steps 5 and 6 of data analysis) is widely used for time-frequency analysis of non-stationary signals, but it has limitations: moderate spectral resolution, a time–frequency trade-off, and high variance and leakage unless averaged.

### Potential solution

The Multitaper method addresses these issues by using multiple orthogonal tapers to reduce variance and leakage while preserving frequency resolution. This results in more accurate and stable spectral estimates, making it ideal for stationary signals where high-resolution spectra are needed. While the multitaper method sacrifices time resolution, it provides superior noise reduction and spectral precision compared to Short FFT. Below is the code to use multitaper analysis:

> filename='UPEs.mat';

>load (filename)

>names=fieldnames(UPEs);

>fs=20;

>minF=0.001;

>maxF=fs/2;

>frequency_range=[minF maxF];

>taper_params=[3 5];

>window_params=[10 2];

>min_nfft=0;

>detrend_opt='off';

>weighting='adapt';

>plot_on=false;

>verbose=false;

>numSubjects=20;

>for n=1:size(names,1)

> field=names{n,1};

> for i=1:numSubjects

> trace=UPEs.(field){:,i};

> [spect,stimes,sfreqs] = multitaper_spectrogram(trace,…,

> fs,frequency_range,taper_params, window_params,…,

> min_nfft, detrend_opt, weighting, plot_on, verbose);

> subfieldName=[field num2str(i)];

> Spectrogram.(subfieldName).power=nanpow2db(spect);

> Spectrogram.(subfieldName).frequency=sfreqs;

> Spectrogram.(subfieldName).time=stimes;

> end

>end

***Note:*** Parameters specifying the input data are 1) filename: the name of the structure variable; 2) fs: sampling frequency; 3) minF: minimum frequency of interest; 4) taper_params: time-half bandwidth product and number of tapers. The time-half bandwidth product (TW) controls the trade-off between frequency resolution and spectral leakage in multitaper analysis. A common choice is TW = 2–4, to balance resolution and stability. The number of tapers (K) is typically 2×TW − 1, ensuring orthogonality and optimal variance reduction. For shorter signals or when higher frequency resolution is needed, choose a lower TW; for more stable estimates and longer signals, a higher TW and more tapers are appropriate; 5) window_params: window size (seconds) and step size (seconds); 6) min_nfft: minimum allowable NFFT size.; 7) detrend_opt: how each time windowed segment of your signal is detrended before computing the spectrum. This can have a meaningful impact on the spectral estimates, especially in low-frequency ranges or when dealing with nonstationary signal. Options include: 7a) Linear -Removes a best-fit straight line from each segment. It removes linear trends without distorting oscillatory components; 7b) Constant - Removes the mean (i.e., DC offset) from each segment. This is useful if linear drifts are not a concern but want to eliminate DC bias. Reduces power at 0 Hz (DC) but leaves linear trends intact. It may still cause low-frequency artifacts; 7c) OFF - Preserves all trends. It can inflate low-frequency power and increase variance in spectral estimates. Use only if you’re certain the data is already stationary or if you intentionally want to preserve any baseline shifts; 8) weighting: determines how to combine the individual spectra obtained from each taper. Options include: 8a) Unity- Each taper is given equal weight (i.e., simple average); May be suboptimal if some tapers capture more signal energy than others; 8b) Eigen - Weights are proportional to the eigenvalues of the tapers (energy concentration in frequency band); Helps reduce bias slightly, especially when only a few tapers have high energy concentration; 8c) Adapt - Adaptive weighting — weights are adjusted based on signal content to minimize variance; especially useful when signal contains transient components. Noise levels are high and there are strong oscillations that may leak across frequencies. 9) plot_on: plot the spectrogram if true; 10) verbose: display spectrogram properties if true.; 11) numSubjects: number of subjects.

### Problem 2

The design of the filter impacts the result of DFA analysis (see Step 7 of data analysis).

### Potential solution

**Figure 4:**
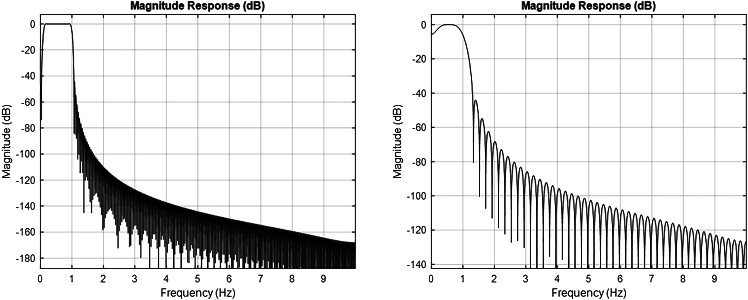
Frequency response of a filter The quality and type of filter impact the computation of the DFA scaling exponent. Accordingly, attention should be dedicated at the selection of appropriate filters. **Left**: In the 0.1–1 Hz range, the response is nearly flat, ensuring minimal distortion of signals in this band. The transition from the passband to the stopband is gradual, with no noticeable oscillations or ripples. This indicates that the filter provides consistent gain across the desired frequencies while smoothly attenuating unwanted higher frequencies. **Right**: In the 0.1–1 Hz range, the response shows pronounced fluctuations in gain, indicating variability in signal amplification across this band. Beyond 1 Hz, the response exhibits a sharp transition into the stopband, where the oscillations persist but gradually decay. Overall, the left graph represents a filter with superior performance for preserving the 0.1–1 Hz range due to its flat response and lack of ripple. Filter has been built with the following line in MATLAB: designedFilter = fir1(50/hp, [filter_order, lp/(fs/2)], ‘bandpass’, hann(filter_order + 1), ‘scale’). For the right filter, filter_order=50/hp, for the left filter, filter_order=10/hp. hp=0.1; lp=1; fs=20 (hp: high-pass filter, in Hz; lp= low-pass filter, in Hz; fs=sampling frequency, in Hz). The code is used for example in ComputeDFA_UPE_singleWindow.mat.

When selecting a bandpass filter, it is helpful to visualize the frequency response graph, which shows how the filter affects different frequency components of the signal. A proper bandpass filter should 1) exhibit a flat and wide passband within the desired frequency range (0.1 and 1 Hz in the example), 2) minimally distort of the target frequencies, 3) providing sharp roll-offs at the lower and upper cutoff frequencies to effectively attenuate unwanted frequencies. Finally, the filter should have a smooth passband with minimal fluctuations (low ripple) and strong suppression of frequencies outside the target range (good stopband attenuation) to effectively reduce noise and interference. Examples of good and bad pass filters are provided in Figure 4.

### Problem 3

To apply DFA, the region should be linear, and the designed filter should not have introduced unwanted correlation (see Step 7 of data analysis).

### Potential solution

**Figure 5:**
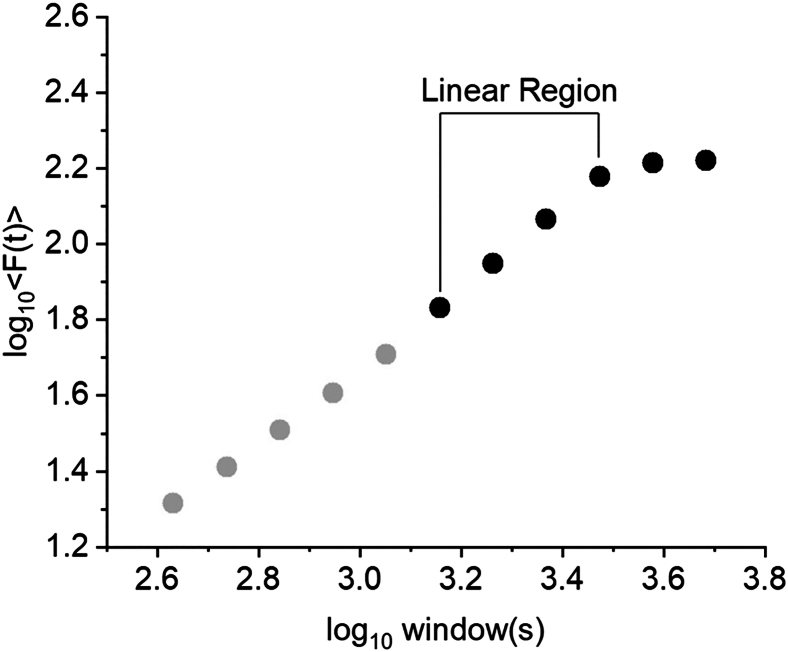
DFA scaling exponent The filters applied to the DFA pipeline distort the temporal correlations of the signal. Accordingly, it is important to identify the windows where the filter distorted the signal. White noise traces are generated and used to identify the impact of the filtering process on the temporal correlation. Example of a graph showing in gray the region in which white noise traces have a value of the DFA scaling exponent different from 0.5 (indicating distortions introduced by filtering); this region cannot be used for analysis. In the graph the region to use for computing DFA scaling exponent is indicated (linear region) and represented the linear region of the graph in which the white noise traces had a scaling exponent of 0.5 (black dots).

To select the proper FitInterval for DFA, it is important to look first for a linear portion of the graph. In addition, in that same interval, white noise traces should have a DFA scaling exponent of about 0.5, indicating no correlation. If the designed filter introduced unwanted correlation, that will be apparent because the white noise traces would have DFA scaling exponent different from 0.5. Example is provided in Figure 5.

### Problem 4

The decision of the mixed-effect model formula (Statistical section) depends on the structure of the data, experimental design and questions (see Section on quantification and statistical analysis).

### Potential solution

Selecting the most appropriate mixed-effects model for a given experimental design involves a balance between theoretical justification, data structure, and model performance. While including more random effects leads to a more realistic representation of the data, it also increases the risk of model convergence issues or singular fits, especially when the number of observations per subject is limited. Therefore, model selection should balance theoretical justification with practical feasibility, ensuring that the complexity of the random effects structure is supported by the available data. Given 20 subjects performing 5 tasks under 3 electrode placements—resulting in repeated measurements per subject — it is essential to account for within-subject dependencies using random effects. A good starting point is to define the fixed effects based on your research questions: whether you expect differences across electrodes, tasks, or their interaction, accounting for repeated measures. This typically results in a formula such as Score ∼ Electrode ∗ Task + (1 | Subject). Then, gradually build complexity by adding crossed random effects, such as (1 | Subject:Electrode) and (1 | Subject:Task), which allow for baseline variability depending on the specific electrode-task-subject combination. If convergence allows, consider including random slopes, such as (Task | Subject:Electrode), which permit the effect of task to vary across subject-electrode pairings—useful when you suspect that task performance interacts with both individual differences and electrode placement. After fitting candidate models, assess convergence and check for singular fits using isSingular(model). Singular models often indicate overfitting and may require simplification of the random effects structure. You can also inspect the variance components with VarCorr(model) to identify random effects that contribute little to explaining variability (e.g., near-zero variances or extreme correlations). Model comparison can be done using likelihood ratio tests (anova(model1, model2)) for nested models or information criteria like AIC and BIC, where lower values indicate better fit relative to complexity. Ultimately, the best model should reflect the experimental design, converge reliably, and not be overly complex for data available. See code below for comparison between different models:

># Linear Mixed Models Comparison Script

library(lme4)

library(lmerTest)

library(tidyverse)

file_path <- " "

dataset <- readxl::read_excel(file_path)

dataset$Subject <- as.factor(dataset$Subject)

dataset$Electrode <- as.factor(dataset$Electrode)

dataset$Task <- as.factor(dataset$Task)

formulas <- list(

 "Model 1" = Entropy ∼ Electrode + Task + (1 | Subject),

 "Model 2" = Entropy ∼ Electrode ∗ Task + (1 | Subject),

 "Model 3" = Entropy ∼ Electrode ∗ Task + (1 | Subject) + (1 | Subject:Electrode),

 "Model 4" = Entropy ∼ Electrode + Task + (1 + Task | Subject),

 "Model 5" = Entropy ∼ Electrode ∗ Task + (1 | Subject) + (1 | Subject:Task)

)

models <- list()

valid_model_names <- c()

model_aic <- c()

model_bic <- c()

model_loglik <- c()

for (i in seq_along(formulas)) {

 formula <- formulas[[i]]

 model_name <- names(formulas)[i]

 cat(paste0("Fitting ", model_name, ": ", deparse(formula), "\n"))

 tryCatch({

  model <- lmer(formula, data = dataset)

  if (isSingular(model)) {

  warning(paste0("Model '", model_name, "' is singular. Consider simplifying the random effects structure."))

  } else {

  models[[model_name]] <- model

  valid_model_names <- c(valid_model_names, model_name)

  model_aic <- c(model_aic, AIC(model))

  model_bic <- c(model_bic, BIC(model))

  model_loglik <- c(model_loglik, logLik(model))

  }

 }, error = function(e) {

  warning(paste0("Model '", model_name, "' failed to fit: ", e$message))

 })

}

if (length(models) > 0) {

 comparison_table <- tibble(

 Model = valid_model_names,

 AIC = model_aic,

 BIC = model_bic,

 LogLik = model_loglik

 ) %>%

 arrange(AIC)

 cat("\nModel Comparison Table (sorted by AIC):\n")

 print(comparison_table)

## Resource availability

### Lead contact

Further information and requests for resources should be directed to and will be fulfilled by the lead contact, Nirosha J. Murugan (nmurugan@wlu.ca).

### Technical contact

Technical questions on executing this protocol should be directed to and will be answered by the technical contact, Mattia Bonzanni (mab4092@med.cornell.edu).

### Materials availability

This study did not generate new unique reagents.

### Data and code availability

The datasets and codes generated during this study are available at Zenodo: https://doi.org/10.5281/zenodo.15830349.

## Acknowledgments

The authors acknowledge support from the National Sciences and Engineering Council of Canada (NSERC), Discovery Grant RGPIN-2021-03783 (to N.J.M.), as well as the New Frontiers in Research – Exploration Program, grant NFRFE-2020-01351 (to N.J.M.), and the Optica Foundation. N.R. and N.J.M. acknowledge support from the Allen Discovery Center at Tufts University.

## Author contributions

M.B.: analysis and interpretation of the results, draft manuscript preparation, and revision. N.R.: study conception and design, analysis and interpretation of the results, draft manuscript preparation, revision, and funding acquisition. N.J.M.: study conception and design, analysis and interpretation of the results, draft manuscript preparation, revision, funding acquisition, and project administration.

## Declaration of interests

The authors declare no competing interests.
